# Sulfonic Acid-Functionalized SBA-15 as Strong Cation-Exchange Sorbent for Solid-Phase Extraction of Atropine and Scopolamine in Gluten-Free Grains and Flours

**DOI:** 10.3390/foods9121854

**Published:** 2020-12-12

**Authors:** Lorena González-Gómez, Judith Gañán, Sonia Morante-Zarcero, Damián Pérez-Quintanilla, Isabel Sierra

**Affiliations:** Departamento de Tecnología Química y Ambiental, E.S.C.E.T, Universidad Rey Juan Carlos, C/Tulipán s/n, Móstoles, 28933 Madrid, Spain; lorena.gonzalez@urjc.es (L.G.-G.); judith.ganan@urjc.es (J.G.); sonia.morante@urjc.es (S.M.-Z.); damian.perez@urjc.es (D.P.-Q.)

**Keywords:** tropane alkaloids, strong cation-exchange solid-phase extraction, mesostructured silicas, gluten-free, cereals, pseudocereals, legumes, HPLC-MS/MS

## Abstract

A novel method was developed and applied to the determination of the most representative tropane alkaloids (TAs), atropine and scopolamine, in gluten-free (GF) grains and flours by HPLC-MS/MS. Accordingly a suitable sample treatment procedure based on solid-liquid extraction (SLE) and followed by strong cation-exchange solid-phase extraction (SCX-SPE) was optimized. SBA-15 mesostructured silica functionalized with sulfonic acids was evaluated as sorbent. The proposed method was fully validated in sorghum flour showing good accuracy with recoveries in the range of 93–105%, good linearity (R^2^ > 0.999) and adequate precision (RSD < 20%). Low method quantification limits (MQL) were obtained (1.5 and 2.4 µg/kg for atropine and scopolamine, respectively) and no matrix effect was observed thanks to the extraction and clean-up protocol applied. The method was applied to 15 types of GF samples of pseudocereals (buckwheat, quinoa and amaranth), cereals (teff, corn and blue corn, sorghum and millet) and legumes (red and green lentil, chickpea and pea). Atropine was found above the MQL in eight of them, with values between 7 and 78 µg/kg, while scopolamine was only found in teff flour, its concentration being 28 µg/kg. The method developed is an interesting tool for determining TAs in a variety of samples of GF grains and flours.

## 1. Introduction

Nowadays, gluten-free (GF) products are one of the food and beverages marketed with best growth projection, as gluten must be eliminated from the diet of people suffering from celiac disease [1,2]. In addition, the development of novel tasty and healthy food products prepared with them is an attractive trend for the food industry because of the potential functional properties of some GF grains and flours (rich in vitamins, minerals, antioxidants, fiber, etc.) [2,3]. For example, amaranth, buckwheat, chickpea, corn, millet and quinoa flours have been evaluated on GF bread production as wheat flours substitutes [1,4]. To improve its nutritional quality, GF cakes, cookies, pasta and snacks have been successfully produced from sorghum, millet, pea and lentil flours [5,6,7]. In recent years, the interest in teff has also increased noticeably, making it a suitable and alternative product to prepare GF foods nutritionally improved [8]. Therefore, due to the absence of gluten in the grain and to the very attractive nutritional profile, some GF cereals, pseudocereals and legumes are experiencing a renewed commercial interest, which emphasizes the need to carefully control the occurrence of tropane alkaloids (TAs) in this kind of samples.

TAs are secondary metabolites produced by a broad variety of plants from families such as *Convolvulaceae, Brassicaceae, Cruciferae,* among others, being more abundant in the plant family *Solanaceae* [9]. There are more than 200 compounds found at low concentration, atropine and scopolamine being the best-known representatives of this type of alkaloids [10]. In recent years, there has been a growing interest in these natural toxins due to their presence as contaminants in food and feed, as they are a potential hazard for human and animal health [9]. Stramonium seeds (*Datura stramonium*) are usually the most frequent cause of the contamination, due to their similar size and to the maturation at the same time with crops like cereals, pseudocereals and legumes with the same natural cycle [11]. This may be especially worrying in organic agriculture since a nonchemical weed control can lead to increase in-field presence of these potentially dangerous plants between the crops [12].

The emergence of multiple Rapid Alert System Feed and Food (RASFF) notifications in recent years [13] has highlighted the need for a more accurate knowledge on the presence of TAs in commercial foods. As a result, sensitive and reliable analytical methods, suitable for the quantification of TAs in different matrices, especially in food from plant origin, have been developed to provide adequate data to support the estimation of actual TAs occurrence in the diet. Nowadays, HPLC-MS/MS is the recommended technique [14] and one of most widely employed to detect trace levels of TAs in foods, thanks to its great sensitivity and to its capability for structural elucidation, which enables unequivocal identification of compounds. However, the complexity of the food samples may lead to the introduction of interferences into the chromatography system, which can induce matrix effects.

A difficult task for the determination of TAs in foods is the development of a suitable sample-treatment procedure. Regarding the extraction step, solid-liquid extraction (SLE) is the most common technique for cereals and similar foods, using acidified water or a mixture of organic solvents with acidified water [11,15,16]. Nevertheless, because of the complexity of these kinds of matrices, which contain many components with different physicochemical properties, such as, proteins, sugars, fats, etc., an additional clean-up step is usually required to remove interferences, as well as to improve the sensitivity and accuracy of the method [17,18]. On the other hand, the development of new materials for their application as sorbents in sample preparation is another challenge in analytical chemistry, as these materials usually have a remarkable role in preconcentration task and selective extraction [19,20,21,22]. For example, mesostructured silicas have acquired increasing research interest in sample preparation and have been evaluated as a good alternative to conventional sorbents, such as polymeric materials and amorphous silica [23,24,25]. This is because of their desirable characteristics such as highly ordered and size-controlled mesoporous structure, high surface area, and large pore volume and good chemical stability. Besides, they have high flexibility in functionalization that enables the introduction of hydrophobic, hydrophilic, polar, as well as, charged functional moieties on surface, which could allow an adequate and selective extraction of the target analytes from food matrices.

To the best of our knowledge, mesostructured silicas have not been applied as SPE sorbents in sample preparation for the analysis of TAs [26]. In addition, there are scarce studies were grains or flours free of gluten have been analysed by HPLC-MS/MS [17,18,27,28]. Within this context, the objective of this work was to evaluate sulfonic acid-functionalized SBA-15 mesostructured silicas as strong cation-exchange (SCX) SPE sorbents for the analysis of TAs in GF matrices by HPLC-MS/MS. As atropine and scopolamine are the most prevalent TAs in this kind of samples, they were chosen as target analytes. The new method developed was validated and then applied to the determination of atropine and scopolamine in 15 samples of pseudocereals (buckwheat, quinoa and amaranth), cereals (teff, corn, sorghum and millet) and legumes (lentil, chickpea and pea), to provide a survey of the presence of both TAs in GF grains and flours.

## 2. Materials and Methods

### 2.1. Reagents and Materials

Tetraethylorthosilicate (TEOS) 98% and poly (ethylene glycol)-block-poly (propylene glycol)-block-poly (ethylene glycol) (EO20PO70EO20, Pluronic 123, P123) were purchased from Sigma-Aldrich (St. Louis, MO, USA). Acetonitrile (ACN) and methanol (MeOH) HPLC-MS grade, HCl 37%, acetic acid (HAc), H_2_O_2_ 30%, ammonia solution 32% and NaOH were obtained from Scharlab (Barcelona, Spain). (3-mercaptopropyl) triethoxysilane (MPTES) 94% was from Alfa Aesar (Karlsruhe, Germany). Formic acid (HCOOH) HPLC-MS grade was from Fluka (Busch, Switzerland). NaCl was purchased from Panreac Química (Castellar del Vallès, Bacerlona, Spain). Ultra-pure water (resistance 18.2 MΩ cm) was obtained from a Millipore Milli-Q-System (Billerica, MA, USA). Polyethylene frits (0.20 µm), filter membranes of nylon (0.45 µm), empty syringes (3 mL) and nylon syringes filters (0.45 µm) were purchased from Scharlab (Barcelona, Spain). MFE-PAK^®^ SCX SPE commercial cartridges with sulfonic-acid functionalized amorphous silica as sorbent were purchased from Análisis Vínicos (Tomelloso, Spain).

The TAs standards used were of high-purity grade >98%. Scopolamine hydrobromide and atropine sulfate were obtained from Sigma-Aldrich. Stock standard solutions (1000 mg/L) were prepared by diluting in MeOH suitable amounts of each TA and stored at −20 °C in the dark. A working standard solution containing both TAs was prepared at the desired concentration in ACN/H_2_O (50:50, *v*/*v*) and stored at −20 °C in the dark.

### 2.2. Samples

Fifteen commercial products, four samples of GF grains and eleven samples of GF flours, were purchased from local supermarkets located in Madrid (Spain). The samples analysed were pseudocereals (buckwheat and buckwheat flour, quinoa and amaranth), cereals (teff flour, refined corn flour, corn flour, blue corn flour, sorghum flour and peeled millet) and legumes (green and red lentil flours, chickpea flour and pea flour). Before extraction, grain samples were milled to obtain a fine powder for their homogenization. Samples were stored in the dark at room temperature until their analysis. The samples were codified by indicating in the first letters the type of the product, followed by an F (for flours) or a G (for grains) and, additionally, depending on their type of farming an O (for organic) and C (for conventional). Finally, a number was added to the code for similar samples but with different brands (see Table S1 in supplementary material). For each sample, information declared in the nutrition facts label is recorded in Table S2.

### 2.3. Synthesis of Mesostructured Silicas

SBA-15 was prepared following the method proposed by Zhao et al. [29]. Firstly, 19.36 g of P123 were dissolved in 576 mL of 2 M HCl and 144 mL of distilled water. The mixture was stirred in a round bottom flask at 35 °C in a silicone bath until P123 was completely dissolved in the HCl solution. Then, 40.8 g of TEOS were added drop by drop and stirred for 20 h. After 20 h, the stirring was stopped, and the temperature was raised to 80 °C and it was left 24 h at this temperature to carry out an ageing process. The material was collected by filtration, washed with distilled water, air-dried and calcined (8.5 h of ramp up to 500 °C and 12 h at 500 °C).

Sulfonic acid-functionalized SBA-15 silicas were prepared according to Yang et al. [30] using a simple two-step synthesis route, involving first thiol functionalization and subsequent oxidation to sulfonic acid groups. Briefly, 2.5 g of SBA-15 were dissolved in 250 mL of 0.1 M HCl aqueous solution and MPTES was then added in different molar ratios of MPTES/SiO_2_ (0.05, 0.10 and 0.18). After stirring for 7 h at room temperature, the mixtures were transferred to an autoclave and remained at 100 °C for 24 h. The solid products were filtered and washed with Milli-Q water and dried at 50 °C overnight. After that, the silicas were suspended in 335 g of 2 M HCl and 11.4 g of H_2_O_2_ (30%) were added. The mixtures were stirred 5 min at room temperature and then were transferred into an autoclave keeping them at 100 °C during 6 h. The resulting materials (denoted as L-SBA-15-SO_3_^−^, M-SBA-15-SO_3_^−^ and H-SBA-15-SO_3_^−^, for the low, L, medium, M, and high, H, MPTES/SiO_2_ ratio, respectively) were recovered by filtration and washed with Milli-Q water.

### 2.4. Characterization and Evaluation of Mesostructured Silicas

A Philips diffractometer (model PW3040/00 X’Pert MPD/MRD at 45 KV and 40 mA) was used to obtain X-ray diffraction (XRD) patterns of the silicas, using Cu-Kα radiation (λ = 1.55418 Å). N_2_ gas adsorption-desorption isotherms were recorded using a Micrometrics ASAP 2020 analyzer. The Brunauer–Emmett–Teller (BET) method was used to calculate the surface specific area (S_BET_) and the Baret–Joyner–Halenda (BJH) model on the desorption branch was used to obtain the pore size distribution. An XL30 ESEM Philips microscope with an energy dispersive spectrometry system was used to obtain Scanning Electron Micrographs (SEM) of the materials. ^13^C cross-polarization magic angle spinning nuclear magnetic resonance (^13^C CP-MAS-NMR) and pulse decoupling angle ^29^Si solid-state nuclear magnetic resonance (^29^Si-PDA-MAS-NMR) spectra were recorded on a Bruker Avance III/HD 9.4 Teslas Spectrometer operating at 400 MHz proton frequency. A Flash 2000 Thermo Fisher Scientific Inc. analyzer was used to perform elemental analysis (EA) of sulfur (% S).

In order to verify the cation-exchange capacity of the sulfonic acid-functionalized SBA-15 silicas, an acid-base titration was carried out according to Margolese et al. [31]. In a typical experiment, 0.05 g of material were added to 10 g of 2 M NaCl. The suspension was stirred for 1 h and thereafter titrated with 0.01 M NaOH employing phenolphthalein as an indicator. Finally, the synthesized materials were evaluated as SCX-SPE sorbents toward the target TAs. For this purpose, 50 mg of L-SBA-15-SO_3_^−^, M-SBA-15-SO_3_^−^ and H-SBA-15-SO_3_^−^ were packed in 3 mL SPE cartridges to be evaluated against a standard solution of TAs (0.5 mg/L). MFE-PAK^®^ SCX commercial sorbent (50 mg) was also evaluated, following the protocol provided by the manufacturer. Accordingly, standards were dissolved in 4 mL of MeOH/0.025 M phosphate buffer pH 4.8 (50:50, *v*/*v*) solution and loaded onto the cartridges, previously conditioned with 2 mL of MeOH and 2 mL of 0.025 M phosphate buffer pH 4.8. The cartridges were washed with 2 mL of 0.025 M phosphate buffer pH 4.8 and TAs were eluted with 3 × 3 mL of MeOH containing 10% ammonia solution (pH 11.8).

### 2.5. Optimized SLE-SPE Sample Extraction Procedure

**Figure 1:**
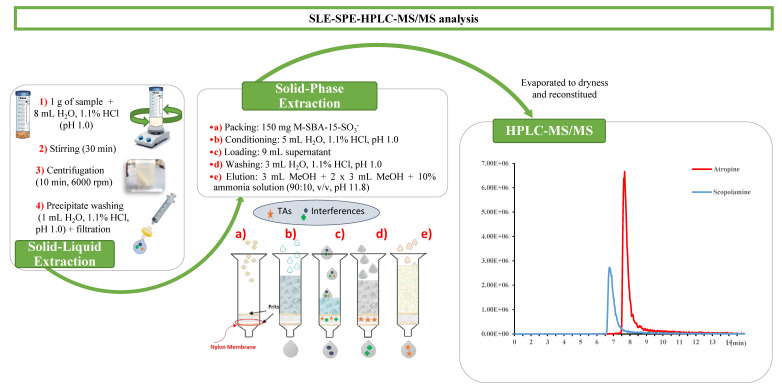
Schematic representation of the optimized extraction procedure and chromatographic analysis.

Approximately 1 g of sample was weighed in a 50 mL Falcon tube with an accuracy of 0.0001 g and 8 mL of water acidified with 1.1% HCl (pH 1.0) were added. The mixture was stirred for 30 min at room temperature and then was centrifuged with a Rotofix 32A centrifuge (Hettich, Germany) at 6000 rpm for 10 min. The supernatant was recovered and the precipitate was washed with 1 mL H_2_O, 1.1% HCl (pH 1.0). The supernatant obtained was mixed with the previous one and filtered through a 0.45 µm nylon filter, prior to the purification step by the SPE procedure. Empty cartridges were packed with 150 mg of M-SBA-15-SO_3_^−^ material and plugged with frits of polyethylene at both ends. A nylon membrane (0.45 µm pore size) was also inserted at the bottom of the sorbent bed to prevent the loss of material during the sample loading. Cartridges were preconditioned with 5 mL of acidified aqueous solution (1.1% HCl, pH 1.0) at a flow rate of 1 mL/min. Once the supernatant was loaded, cartridges were dried with a Scharlab ExtraVac^®^ solid extraction vacuum manifold 12 port (Scharlab, Barcelona) connected to a vacuum pump at 10 psi, and then washed with 3 mL water acidified with 1.1% HCl (pH 1.0) to remove interferences. Then, the cartridge was dried for 1 min. Finally, the target analytes were eluted with 3 mL of MeOH followed by 2 × 3 mL MeOH containing 10% of ammonia solution (pH 11.8). During elution, the first drops were allowed to pass through the cartridge in each elution and the valve was closed for 5 min. Eluates were evaporated to dryness and redissolved with 500 µL of ACN/H_2_O (50:50, *v*/*v*) for analysis in the chromatography system (Figure 1).

### 2.6. Chromatographic Analysis and Instrumental Parameters

For the chromatographic analysis, a Varian 1200/1200 L LC-MS/MS was used (Varian Ibérica, España) with a ProStar 410 autosampler (100 μL loop), two solvent deliver module ProStar 210/215, a thermostatic compartment for the column and a triple quadrupole mass spectrometer (1200 L TQ) with electrospray ionization (ESI) ion source (data acquisition system MS Workstation version 6.3). A C18 Kromaphase 100 column (150 mm × 2.0 mm, 3.5 μm particle size) at 30 °C was used for chromatographic separation with gradient elution (eluent A—0.1% HCOOH in ACN; eluent B—0.1% HCOOH in Milli-Q water). The gradient started at 10% A and 90% B, then eluent A increased linearly to 70% in 10 min and returned to initial conditions in 1 min. This composition was held for 4 min. The total run-time of the method was 15 min. The flow rate was set at 0.25 mL/min and the injection volume was 10 µL. The column was equilibrated with 10% A for 1 min before to the next run.

Mass spectrometry acquisition was performed using ESI ion source operating in positive mode. N_2_ was used as both drying and nebulizer gas and argon was set as collision gas under the following conditions: The N_2_ drying gas was set at 350 °C and 22 psi, nebulizer gas pressure was set at 58 psi; the capillary voltage was held at −5000 V and shield at −600 V. Collision gas was set at 1.90 mTorr and detector voltage at 1480 V. The mass spectrometric fragmentation was optimized for each compound. Multiple reaction monitoring (MRM) mode was used for all analytes (mass peak width Q1i 2.5; mass peak width Q3 2.5; scan width in MRM 2 s). Compounds were monitored at cone voltage of 70 V with the following transitions: 290 → 91.1 (CE = 34 V), 290 → 93.0 (CE = 28 V) and 290 → 124.0 (CE = 22 V) for atropine; 304 → 121.1 (CE = 20 V), 304 → 138.0 (CE = 16 V) and 304 → 156.0 (CE = 14 V) for scopolamine.

Standard solutions were prepared to evaluate the instrumental parameters in the HPLC-MS/MS. These parameters were linearity, precision, detection (LOD) and quantification (LOQ) limits. Linearity was evaluated between 0.005 and 25 µg/mL. Repeatability and reproducibility were evaluated at four concentration levels (the lowest concentration near to LOQ). Repeatability was carried out analyzing each standard solution six times, in one day, on the HPLC-MS/MS (n = 6). Reproducibility was evaluated analyzing each standard solution three times on three different days (n = 9). The instrumental limits, LOD and LOQ, were calculated as three and ten times the signal-to-noise ratio (S/N), respectively, corresponding to the lowest standard solution analysed.

### 2.7. Method Validation

The proposed SLE-SPE-HPLC-MS/MS methodology was properly validated in terms of linearity, matrix effects, selectivity, method detection (MDL) and quantification (MQL) limits, accuracy and precision. As there is no official regulation to validate analytical methods for TAs analysis in foods, the validation procedure guidelines established in the SANTE/11813/2017 document for the analytical quality control of pesticides residues in food and feed products [32] and the AOAC Guidelines for Dietary Supplements and Botanicals [33] were followed. Sorghum flour (So-FO) was used as a representative sample for full validation.

Linearity was evaluated through matrix-matched calibration curves that were prepared in three consecutive days by spiking the extract of the sample with an appropriate aliquot of a standard solution containing the TAs to achieve the desired concentration level of the calibration curve. At the same time, a blank sample (unspiked sample) was also extracted for matrix corrections, in case atropine and scopolamine were present in the sample analysed, so their signal can be subtracted. Six known concentration levels, within the linear range evaluated, were used to perform the calibration curves by plotting the analyte concentration against the peak area of each analyte. Linear regression analysis was applied and linearity was expressed as the coefficient of determination (R^2^) and the linearity coefficient (Cm). Cm was calculated as (1 − (SD/average slope)) × 100, where SD is the standard deviation of the calibration slopes obtained on different days. Matrix effect (ME, %) was calculated by comparing the slopes of solvent-based standard calibration curves with matrix-matched calibration curves by means of the following equation: ((slope matrix-matched/slope solvent-based) − 1) × 100), both expressed in the same units. A positive value indicates signal enhancement, whereas a negative value shows signal suppression. Selectivity was demonstrated by identifying the target TAs based on the precursor ion, product ions and retention time (tr) compared to standards (tolerance ± 0.1 min). In positive samples, ion ratios in unit mass resolution MS/MS were verified and should not deviate more than 30% (relative abundance) from the reference value. In blank samples it was verified the absence of signal in the chromatogram. The accuracy was estimated as the mean recovery obtained from three samples for three days (n = 9) of sorghum spiked with the analytes at three concentration levels (the MQL, 5 µg/kg and 100 µg/kg). The concentration level of 5 µg/kg was selected according the European Food Safety Authority (EFSA) recommendation [34] for the analysis of agricultural products, ingredients, food supplements and infusions, to ensure that good recoveries were obtained at this concentration. Recovery values were calculated by comparing the areas of the spiked samples with the areas of simulated samples (samples spiked at the same concentration level but at the end of the extraction procedure prior to their chromatographic analysis). Method precision (expressed as relative standard deviation, RSD%) was assessed in terms repeatability (intraday precision) and reproducibility (interday precision) at the same validation levels as the accuracy assays. Intraday precision was evaluated through the analysis of six replicates on the same day (n = 6), of a sample spiked at each concentration level tested. Interday precision was evaluated through the analysis of three replicate samples throughout three different days (n = 9) and spiked at each validation level. Finally, MDL and MQL was estimated as three and ten times, respectively, the S/N for the chromatographic response in HPLC-MS/MS to the lowest concentration in the matrix calibration.

## 3. Results and Discussion

### 3.1. Characterization of Mesostructured Silicas and Evaluation as SCX-SPE Sorbent

**Figure 2:**
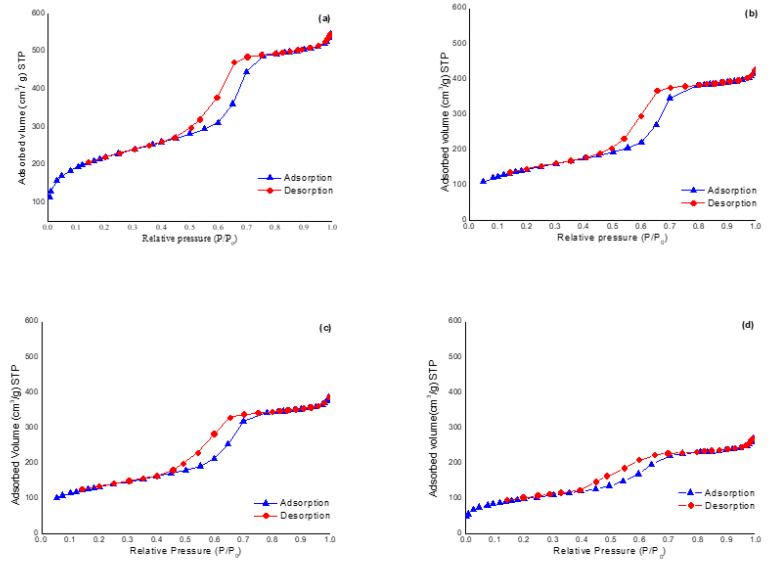
N_2_ adsorption-desorption isotherms of the following materials: (**a**) SBA-15, (**b**) L-SBA-15-SO_3_^−^, (**c**) M-SBA-15-SO_3_^−^ and (**d**) H-SBA-15-SO_3_^−^.

Figure S1 shows XRD patterns of bare SBA-15 and sulfonic-acid functionalized silicas prepared with different MPTES/SiO_2_ molar ratios. XRD pattern of bare SBA-15 exhibits three characteristic Bragg diffraction peaks relative to the (1 0 0), (1 1 0) and (2 0 0) planes at 2θ of 1.04°, 1.66° and 1.93°. This pattern suggests that the silica prepared contains well-ordered hexagonal arrays of one-dimensional channel structure. The decrease in the XRD peaks intensity for sulfonic-acid functionalized silicas provides evidence of functionalization occurring mainly inside the mesopores, as the attachment of organic groups in the mesopore channels tends to reduce the scattering power of the mesoporous silicate wall. The XRD pattern of the sulfonic-acid functionalized SBA-15 also suggests that the well-ordered hexagonal arrangements of the parent structure remained after functionalization. According to the I.U.P.A.C. classification, the N_2_ adsorption-desorption isotherms for these materials (Figure 2) were type IV with an H1 hysteresis loop, which is representative of mesopores. At a relative pressure (*P/P*_0_) of approximately 0.5, the gas volume adsorbed increased, representing capillary condensation of N_2_ within the uniform mesopore structure. The inflexion position shifted slightly toward lower *P/P*_0_ and the volume of N_2_ adsorbed decreased with functionalization. Table 1 shows the physical parameters of N_2_ isotherms, such as S_BET_, BJH average pore diameter and total pore volume for mesostructured silicas. Bare silica possessed very high S_BET_ (780 m^2^/g), a pore volume of 0.80 cm^3^/g and a BJH pore diameter of 56 Å, typical of surfactant-assembled mesostructures. After functionalization, with the increase of the MPTES/SiO_2_ molar ratio, a decrease in the S_BET_ (from 587 to 350 m^2^/g), pore volume (from 0.7 to 0.4 cm^3^/g) and BJH average pore diameter (from 55 to 36 Å) was observed (Table 1), that can be easily interpreted as the presence of pendant group on the surface that partially blocks the adsorption of N_2_. The narrow pore size distributions found for functionalized silicas attest their uniform framework mesoporosity (Figure S2). On the other hand, as evidenced in the SEM micrographs, the SBA-15 material prepared showed uniform particles with cylindrical shape (1 µm in one axis and 500 nm in the other, on average) and functionalization did not affect the morphology of the particles.

**Figure 3:**
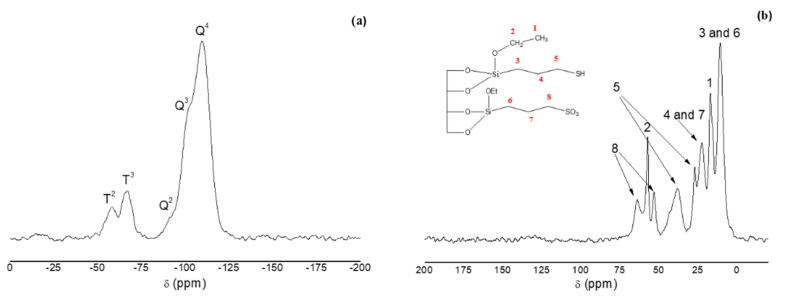
(**a**) ^29^Si PDA-MAS-NMR and (**b**) ^13^C CP-MAS-NMR spectra of H-SBA-15-SO_3_^−^.

Figure 3a shows the ^29^Si PDA-MAS-NMR spectrum of H-SBA-15-SO_3_^−^.The two main peaks appear at −109 and −102 ppm, which are assigned to Q^4^ framework silica sites ((SiO)_4_Si) and Q^3^ silanol sites ((SiO)_3_SiOH), respectively. The spectrum also shows, as a shoulder, the Q^2^ peak ((SiO)_2_Si(OH)_2_) at −91 ppm. The small value of the Q^2^ sites is related to materials which display a good degree of condensation. At −67 and −58 ppm, two other peaks appeared that are assigned to T^2^ ((SiO)_2_SiOH–R) and T^3^ ((SiO)_3_Si-R) sites, respectively, which verify the anchoring of the organic ligand to the silica. The ^13^CP-MAS-NMR spectrum of H-SBA-15-SO_3_^−^ (Figure 3b) shows information respect to the groups anchored on the SBA-15 surface that confirms its functionalization. As can be seen in Figure 3b, the three carbon atoms related to the propyl sulfonic group, numbered (6), (7) and (8), gave signals at 10.4, 22.4, 52.7 and 63.9 ppm, respectively. The signal due to methylene (2) of the ethoxy group appears at 57 ppm and the signal of the methyl group (1) at 16.7 ppm. These results provide evidence that the functionalization was successful. Furthermore, in the spectrum appears additional signals assigned to the carbon atoms (3), (4) and (5), confirming the presence of thiol groups anchored to the pore walls and indicating that part of SH groups could not be oxidized.

The number of functional groups attached to ordered mesoporous silicas was calculated through the sulphur content determined by EA (Table 1). Regarding the % S, it was estimated the presence of 0.5, 0.9 and 1.7 mmol/g of S in the L-SBA-15-SO_3_^−^, M-SBA-15-SO_3_^−^ and H-SBA-15-SO_3_^−^, respectively. However, titration analyses for the determination of SO_3_^−^ groups in the functionalized ordered mesoporous silicas showed a rate of 60%, 56% and 35% for, L-SBA-15-SO_3_^−^, M-SBA-15-SO_3_^−^ and H-SBA-15-SO_3_^−^, respectively, similar results to those are described in the literature [31]. Accordingly, it could be estimated the presence of the 0.3, 0.5 and 0.6 mmol SO_3_^−^/g in L-SBA-15- SO_3_^−^, M-SBA-15-SO_3_^−^ and H-SBA-15-SO_3_^−^, respectively. On the other hand, as it can be seen in Table 1, the recovery percentage for the target analytes in the SPE experiments increased with the functionalization degree of the material.

Regarding the titration results, the SO_3_^−^ content in M-SBA-15-SO_3_^−^ was found to be higher than that in L-SBA-15-SO_3_^−^, as it was expected, which indicated a higher acid capability in M-SBA-15-SO_3_^−^. This could lead to an increase in the active sites available for the retention of TAs, resulting in better extraction efficiency. However, in the H-SBA-15-SO_3_^−^ material, for which sulphur content was found twice that in the M-SBA-15-SO_3_^−^ (1.7 and 0.9 mmol S/g, respectively), the acid capability was similar to M-SBA-15-SO_3_^−^ according to titration results (0.6 and 0.5 mmol SO_3_^−^/g, respectively) and the recovery for TAs was not significantly different in both materials. Hence, M-SBA-15-SO_3_^−^ was chosen for the following experiments to reduce reagent consumption in the synthesis step. In addition, the good recovery results obtained with the M-SBA-15-SO_3_^−^ material were comparable to that obtained with the SCX commercial sorbent, evaluated under similar conditions according to the manufacturer protocol (around 90% in all cases). These results successfully confirmed the potential application of the M-SBA-15-SO_3_^−^ material for TAs extraction in the GF samples.

### 3.2. Optimization of the Sample Treatment

The sample preparation methodology is a very important stage of an analytical process to achieve clean extracts suitable for the measurement step. In this way, a longer life of the HPLC column is achieved and interferences that may hinder the interpretation of results are avoided. For this reason, a sample treatment protocol based on SLE combined with a preconcentration and clean-up stage by SPE was optimized, evaluating the absolute recoveries of TAs in the GF grains and flours.

Firstly, in order to evaluate the extraction capability of the M-SBA-15-SO_3_^−^ sorbent for TAs, different mixtures of polar solvents with a small amount of acid were tested to load the target analytes into the cartridge (Table 2). These media were selected because TAs are more soluble in acid aqueous solutions [33]. Thus, 8 mL of standard solutions of atropine and scopolamine (0.1 mg/L) prepared in: MeOH/H_2_O, 0.5% HAc (2:1, *v*/*v*), ACN/H_2_O, 0.5% HCOOH (3:5, *v*/*v*), ACN/H_2_O, 1% HCOOH (3:5, *v*/*v*) and H_2_O, 1.1% HCl (pH 1.0), were loaded into SPE cartridges packed with 50 mg of M-SBA-15-SO_3_^−^ (as indicated in Section 2.5). The conditioning and washing of the cartridge were performed with 5 mL and 3 mL of the same mixture used for the loading step. As it can be seen in Table 2, with M-SBA-15-SO_3_^−^ as sorbent all recoveries exceeded 70%. The best results were obtained with MeOH/H_2_O, 0.5% HAc (2:1, *v*/*v*) and H_2_O, 1.1% HCl (pH 1.0) with recoveries of 86 ± 3% and 90 ± 7% for atropine and 105 ± 10% and 103 ± 1% for scopolamine, respectively (Table 2). On the other hand, worse recoveries were obtained in all cases when these conditions were assessed with the commercial sorbent (ranging from 15 to 65%).

Besides, experiments were carried out using two different samples, Bu-F1O (buckwheat flour) and So-FO (sorghum flour). In this case, 1 g of sample was subject to SLE (as indicated in Section 2.5) with 8 mL of the same mixture of solvents and then the SPE step was applied using 50 mg of M-SBA-15-SO_3_^−^ as sorbent. Samples spiked before analysis with 0.1 mL of a 1 mg/L TAs standard solution, were performed in duplicate and compared with a simulated sample spiked after the SPE process (and before evaporation). The recoveries were estimated by comparison of the areas of the spiked samples with the areas of the simulated samples. The acidified aqueous medium (H_2_O, 1.1% HCl, pH 1.0) was selected as the extraction solvent, as it provided the best TAs recoveries in both samples (Table 2). On the other hand, when organic solvents (MeOH or ACN) were used, very low recoveries were observed (in general less than 16%). As TAs contain a ternary amine that is protonated at low pH, the good solubility of the atropine and scopolamine in H_2_O enables a selective extraction with aqueous acid, excluding nonpolar compounds [34]. Furthermore, in comparison with the other extraction media evaluated, the use of acidified water is cheaper and safe. Better recoveries were obtained in the So-FO sample (66 and 48% recovery for atropine and scopolamine, respectively) than in the Bu-F1O (43 and 25%, respectively). This fact may be due to the nutritional composition of buckwheat flour, rich in minerals, which can also interact with the SPE sorbent [35].

As plenty of matrix compounds from GF samples were leached into the raw extracts when acidic water was used during the TAs extraction, other studies were carried out to optimize the SPE conditions and to eliminate the coextracted hydrophilic substances that could interfere with the target analytes. As TAs exist as positively charged molecules in the crude SL extracts, while most of the other interferences are neutral or negatively charged, they can be retained by cation exchange interaction in the sorbent. According to the results shown in Table 3, the M-SBA-15-SO_3_^−^ amount had an important effect on the recovery that significantly increased when the packed amount of sorbent increased from 50 mg to 200 mg, indicating that, in general, 150 mg of this material were the best choice to obtain good recoveries and a clean-up effect. On the other hand, using similar amounts of the commercial SCX sorbent, recoveries were reduced from 40 to 70%.

Additional experiments were carried out to evaluate the effect of the solvent volume in the SLE (H_2_O, 1.1% HCl, pH 1.0) and the type and volume of the elution solvent in the SPE step. These studies were carried out with the Bu-F1O sample, using 150 mg of M-SBA-15-SO_3_^−^ as sorbent. Results obtained indicated that increasing the solvent volume from 5 mL to 8 mL led to a significant increase in the TAs recovery, but no differences were observed between 8 mL and 10 mL, so this volume (8 mL) was used for further experiments. Finally, the SPE elution solvent was optimized, as this solvent should have enough elution capability to desorb the TAs and facilitate their further analysis. Best results were obtained when 3 mL of MeOH followed by 3 × 3 mL of MeOH containing 10% of ammonia solution (pH 11.8) were used for the elution of the compounds, instead of using 3 × 3 mL MeOH with 10% of ammonia solution. These results indicated that a first elution step with an organic solvent could help to break some bonds formed between the analytes and the sorbent, in addition to the subsequent drastic change in pH with the basified organic solvent that disrupts the analyte-sorbent interaction, what results in the complete elution of the basic TAs. No differences were observed in the recovery values between 2 × 3 mL and 3 × 3 mL of MeOH with 10% ammonia solution, so the first one was used for the procedure optimized, as an excess of volume would lead to a long time for the next dryness step.

**Figure 4:**
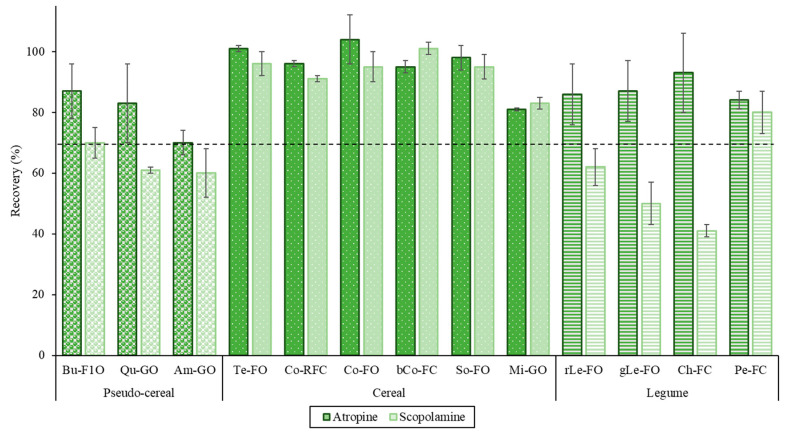
Recovery percentage for atropine and scopolamine in gluten-free (GF) grains and flours under optimized SLE-SPE conditions.

Figure 4 shows the recoveries of atropine and scopolamine obtained from the SLE-SPE procedure optimized, using 150 mg of M-SBA-15-SO_3_^−^ as SCX-SPE sorbent, in the different GF grain and flour samples spiked with the target TAs at 100 µg/kg. As it can be seen, in general, recoveries were higher than 70%, except for scopolamine in quinoa, amaranth, lentil and chickpea flour samples. Knowing that the recoveries improved using a higher amount of sorbent (Table 3), in these samples the use of 200 mg could be a good option to achieve better results.

### 3.3. Optimization of HPLC-MS/MS Conditions and Instrumental Parameters

**Figure 5:**
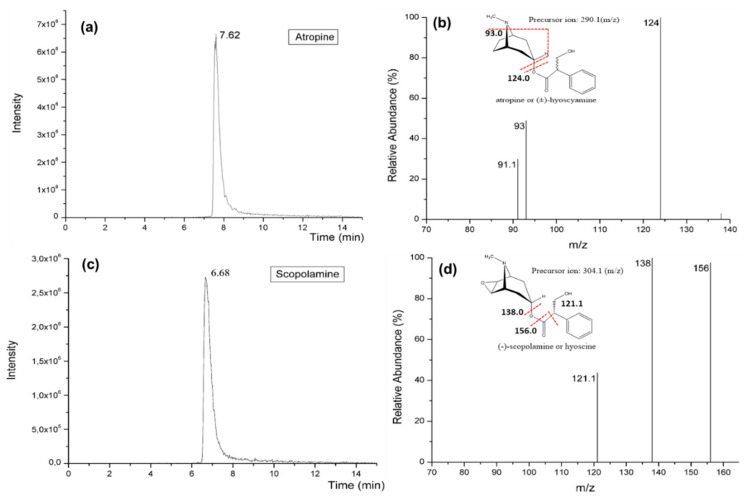
Standard solution mixture of atropine and scopolamine (0.2 µg/mL). (**a**) Extracted ion chromatogram for atropine (m/z 290.1 > 124.0), (**b**) fragment ions mass spectrum of atropine, (**c**) extracted ion chromatogram for scopolamine (m/z 304.1 > 138.0), (**d**) fragment ion mass spectrum of scopolamine.

Figure 5 shows the mass spectrum obtained after optimization of the detection. Different fragments were found using ESI in positive mode. The most common ruptures of TAs are generally produced in the tropane ring. These ruptures generate a characteristic product ion in the case of atropine m/z 124.0 and m/z 138.0 for scopolamine. Another common ion fragment in the case of atropine is m/z 93.0, which is formed by the rupture of the ester and the nitrogen of the tropane ring. Two mobile phases were tested, using 0.1% HCOOH in ACN or 0.1% HCOOH in MeOH as eluent A. In both cases, eluent B was 0.1% HCOOH in Milli-Q water. The best results were obtained using ACN as organic solvent, obtaining good separation and better peak intensity. With this mobile phase, the tr for atropine and scopolamine was found to be 7.62 and 6.68 min, respectively (Figure 5).

Standard working solutions were analysed in the HPLC-MS/MS to evaluate the instrumental parameters. Results are shown in Table S3 (see supplementary material). Linearity was evaluated in a 0.005–5 µg/mL range, with R^2^ > 0.99. Repeatability and within-laboratory reproducibility were in the range 1–4% and 4–8%, respectively, for both analytes. Low LOD and LOQ were obtained: 0.004 and 0.013 µg/L for atropine and 0.014 and 0.05 µg/L for scopolamine, respectively.

### 3.4. Method Performance Parameters

#### 3.4.1. Evaluation of Matrix Effect, Calibrations and Limits

Preliminary studies for investigation of ME on MS/MS detection were carried out. For this purpose, extracts of each sample were spiked at one concentration level (0.1 mg/kg). The peak area for atropine and scopolamine in the spiked samples was compared with that of a standard solution at the same concentration. Results obtained indicated signal suppression for these TAs in some of the tested samples, whereas in some others no ME or a slight increase in the signal was found. This implies that to correctly quantify atropine and scopolamine in some of these samples, it is necessary to use the matrix calibration and not the solvent calibration. In addition, these results confirmed that it was not possible to use a representative matrix for quantification of atropine or scopolamine in all the GF pseudocereals, cereals and legumes evaluated in this work. For this reason, these data were statistically compared by Analysis of Variance (ANOVA) and Duncan’s Multiple range test. As it can be seen in Table S4, three groups of samples were identified, as no significant differences (*p* ≤ 0.05) in the ME were observed between then (group 1: Bu-F1O, Qu-GO, Co-RFC; Co-FO; bCo-FC; Te-FO, Mi-GO and So-FO; group 2: Am-GO, Ch-FC and rLe-FO and group 3: Pe-FC and gLe-FO).

Bearing in mind these results, in order to verify the three groups of samples, matrix-matched calibration curves were prepared by spiking sample extracts of Co-RFC, So-FO, Co-FO and Te-FO (group 1, G1), Am-GO (group 2, G2) and Pe-FC (group 3, G3) with aliquots of a standard solution containing the target TAs. Good linear regression for both compounds was achieved, in all matrices, obtaining R^2^ > 0.993 (Table 4). In addition, the Cm was between 92 and 98% for atropine and 94 and 99% for scopolamine, successfully accomplishing the criteria established on the guidelines (≥92%) [32,33]. The ME was then calculated by comparing the slopes of both matrix-matched and solvent-based calibration curves. The slope values of the matrix-matched calibration curves were significantly lower than the slopes of the solvent-based calibration curves (*p* ≤ 0.05), leading a significant matrix suppression of −35 and −51% for atropine and −51 and −69% for scopolamine in Pe-FC and Am-GO, respectively (Table 4). Therefore, to quantify atropine and scopolamine in samples of the G2 and G3, matrix-matched calibration curves should be used to compensate errors associated with matrix suppression and to perform a true quantification of both compounds. In contrast, almost no ME was found for TAs in the other samples, and no significant differences were observed between the slopes of Co-RFC, So-FO, Co-FO and Te-FO matrix-matched curves. For this reason, since ME was similar for atropine and scopolamine, So-FO can be used as a representative matrix to prepare calibration curves for quantification of TAs in the whole collection of G1 samples. In addition, these results indicated that the extraction protocol developed in this work minimizes the ME compared with other previous works [18,27], allowing one to achieve good MDLs (0.04–0.5 µg/kg and 0.2–1.3 µg/kg for atropine and scopolamine, respectively). On the other hand, as it can be seen in Table 4, MQL for atropine ranged from 0.1 to 1.5 µg/kg and from 0.7 to 4.4 µg/kg for scopolamine, so the method developed offers good limits similar to other previous works. It is important to emphasize that there are not legal limits established for atropine and scopolamine in food and feed (except in cereal-based products for children with a maximum allowable content of 1 µg/kg). The EU is fostering the development of analytical methods with MQL preferably below 5 µg/kg, for atropine and scopolamine in agricultural commodities, ingredients, food supplements and herbal teas [14], that can be achieved with the method developed.

In order to reduce ME and improve accuracy and sensitivity, a preconcentration and clean-up stage by SPE was also applied in other works, using SCX sorbents for the analysis of TAs in cereal and cereal based samples. In this regard, the selection of a suitable material is crucial for SPE method development. Polymeric materials are usually applied as SPE sorbents, but strong hydrophobic interaction on polymeric-based SCX sorbents is not appropriate for the elimination of nonalkaloids, in comparison with silica-based SCX sorbents [36]. For example, Marin et al. [18] tested Strata-X-C and Oasis MCX cartridges, achieving the best recoveries with Strata-X-C (a polymeric cationic mixed-mode sorbent) in buckwheat millet, soy and linseed samples (63–93%). However, in this work even with the SPE purification step and important ME was observed (strong matrix suppression between −60 to −80%) for atropine and scopolamine.

#### 3.4.2. Selectivity, Accuracy and Precision

Positive and noncontaminated (blank) GF samples were used to evaluate the selectivity and to monitor interfering signals at the characteristic tr for the TAs. Firstly, and according to SANTE, it was verified that the tr of both analytes in the sample extracts corresponded to that of the matrix-matched calibration standards, with a SD lower than ±0.1 min. In blank samples no signal was noted in the chromatogram, so it can be concluded that the developed method was selective for the considered TAs. In addition, in positive samples ion ratios in unit mass resolution MS/MS were verified and did not deviate more than 30% (relative abundance) from the value obtained in the corresponding spiked positive samples. Recovery values were calculated by comparing the areas of the spiked samples with the areas of simulated samples. As Table S5 shows, good recovery values for both TAs were obtained at the three levels evaluated, as they were within the range 70–120% as specified in the recommendations [31,32].

The mean recovery values ranged from 93 to 105% (Table S5). On the other hand, as it can be seen in Figure 4, in quinoa amaranth, lentil and chickpea samples spiked at the higher level (100 µg/kg) recoveries lower than 70% were observed for scopolamine. However, this compound could be quantified in these samples using a correction factor, as according to SANTE/11813/2017 document the recoveries between 30 and 70% and 120 and 140% can be acceptable if RSD is ≤20%. These results indicated that the method could be used in these other foods, after confirming the recoveries at the medium and lowest spiking levels. Recoveries obtained in this work were comparable or better (at low concentrations) with those obtained by other authors in previous studies for atropine and scopolamine in grains, flours and related products (50–109%) [17,18,27,28], but the use of internal standards was avoided [15] and lower ME were observed because of the sample extraction procedure applied.

Finally, according to the SANTE recommendations, RSD values for precision should be ≤20% for both intra- and interday precision [32,33]. Therefore, satisfactory results were achieved at the three concentration levels evaluated (Table S5), as the RSD values observed were lower than 11 and 13% for intra- and interday precision, respectively, for atropine and lower than 8 and 19% for intra- and interday precision, respectively, for scopolamine.

#### 3.4.3. Robustness

To assess the method robustness, five different batches of the M-SBA-15-SO_3_^−^ silica were prepared on different days and, after characterization, they were evaluated as SCX-SPE sorbents under the optimized conditions. As shows Table S6, the materials showed very similar S_BET_, pore diameter, pore volume and functionalization degree. After the SLE-SPE-HPLC-MS/MS protocol in Bu-F1O spiked at the higher level (100 µg/kg) with four different batches of sorbent, recoveries obtained were between 85 and 102% for atropine and between 91 and 115% for scopolamine. These recoveries confirmed that the batch of the sorbent has no significant influence on the results.

### 3.5. Application of the Validated Method to Commercial Samples

Finally, to evaluate the applicability the method validated, 15 different GF grains and flour samples of pseudocereals (buckwheat, quinoa and amaranth), cereals (teff, corn and blue corn, sorghum and millet) and legumes (red and green lentil, chickpea and pea) purchased from local markets in Spain were analysed. Each sample was analysed in duplicate. To the best of our knowledge, there is no published data about the presence of the target compounds in some of these samples, such as blue-corn, teff, quinoa, lentil, pea and chickpea.

**Figure 6:**
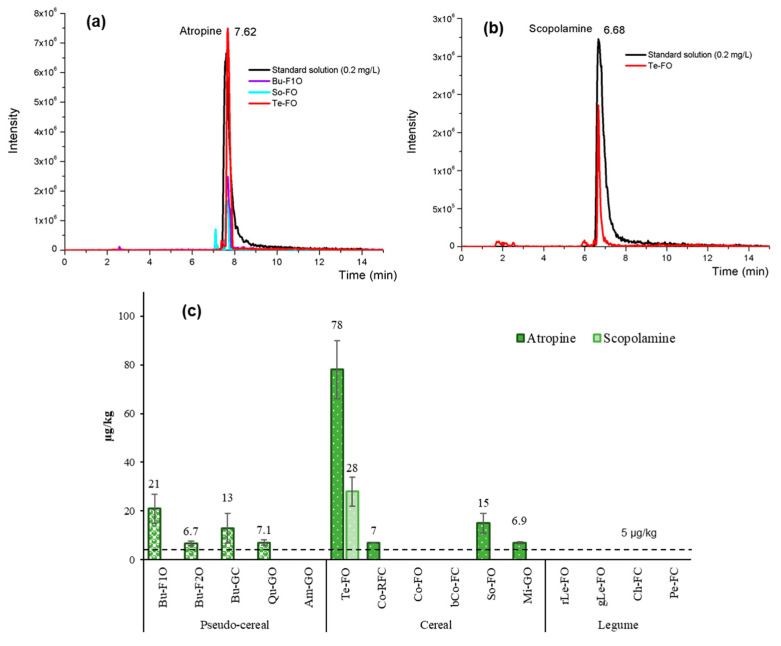
Extracted ion chromatogram of (**a**) atropine (m/z 290.1 > 124.0) and (**b**) scopolamine (m/z 304.1 > 138.0) in a standard solution mixture (0.2 mg/L) and in some positive samples. (**c**) Concentration (µg/kg) of atropine and scopolamine in commercial GF grains and flours.

Figure 6a,b show the chromatograms of the samples with the highest atropine (teff, buckwheat and sorghum flours) and scopolamine (teff flour) content. Results showed in Figure 6c indicated that at least one TA was detected in 60% of the GF samples analysed. Atropine was found above the MQL in eight of the samples, between 6.7 and 78 µg/kg, while scopolamine was only found in Te-FO sample, being its concentration 28 µg/kg. All these samples would exceed the maximum limit set by the Commission Regulation (EU) 2016/239 for cereal-based foods for infants and young children (1 µg/kg) if extended to these type of products [37].

It is well known that ingestion of TAs occurs normally when parts of toxic plants (particularly the seeds) are accidentally mixed with crops of cereals, pseudocereals and legumes during harvest or processing. In this sense, plants of the genus from *Solanaceae* family, which are widely distributed in temperate and tropical regions, can be easily found as weeds in cultivated fields [14]. A high concentration of TAs has been found, for example, in *Datura stramonium, Datura innoxia, Datura. ferox, Atropa belladonna, Atropa mandragora, Hyoscyamus nigrum, Solanum nigurm* and *Brugmasia arborea,* among others. The most common contamination route is related to the coharvesting of weeds sharing the same cultural cycle with specific crops, especially for all those seed-bearing plants that are harvested by mechanical means. *D. stramonium* and *D. ferox* weeds invade some annual crops like buckwheat, sorghum, millet and corn. The weeds ripen with the crop and the weed seeds contaminate the final harvested grain [38]. In this regard, contamination with seeds may not be easily detected by visual inspection, due to similar size, shape and, in some cases, colour (as in the blue corn). This is especially noteworthy as the presence of a single undetected seed per million of crop seeds may pose relevant health risks [27]. For this reason, in 2015 the European Union issued a recommendation on the occurrence of TAs, particularly in buckwheat, sorghum, millet and corn (in that order of priority) and their flours, in addition to other food products as legumes [14]. On the other hand, other GF cereals like teff, traditionally grown as a cereal crop in Ethiopia, are experiencing a commercial interest worldwide. In that respect, it is well reported that in this annual grass crop, the equally toxic immature weeds (mainly *D. ferox*) are cut and dried with the hay causing intoxication in horses [38,39]. As evidence Figure 6c, buckwheat, sorghum, millet, corn and teff samples analysed in this work were all naturally contaminated with TAs (Figure 6c). Besides, all samples of legumes resulted in negative up to the MDL.

Table S7 shows RASFF notifications of the last 5 years (2015–2020) on atropine and scopolamine in cereals and bakery products. As it can be seen, there are fifteen notification related with the presence of these TAs in this kind of foods, and eleven of them were for GF products (i.e., sorghum flour, baking mix and related products based on sorghum, corn, popcorn, corn grits, millet flour, buckwheat flour, soy flakes). In these alerts, atropine was found to be between 4.5 to 1500 µg/kg and scopolamine between 1.77 and 460 µg/kg. In this regard, it is noteworthy to mention that eight GF samples analysed in our work have an atropine amount between 7 ± 1 and 78 ± 12 µg/kg, so all of them exceeded the lowest level notified in the RAFF alerts. In addition, the sample that was positive in scopolamine contained an important amount of this toxic (28 ± 6 µg/kg in teff flour).

The contamination level recorded in sample Te-FO (for the sum of atropine and scopolamine) was higher to 100 µ/kg (intervention threshold set by French authorities for buckwheat flour), so this content cannot be considered negligible from a food safety standpoint (Figure 6c). The Scientific Panel on Contaminants in the Food Chain (CONTAM) of the European Food Safety Authority (EFSA) established an acute reference dose (ARfD) of 0.016 µg/kg body weight, expressed as the sum of atropine and scopolamine [9,40]. On the other hand, in the recent EFSA Scientific Report published about human acute exposure assessment to tropane alkaloids, it was concluded that the main contributors to the coexposure of atropine and scopolamine were bread and other grain milling products [9]. In this sense, 100 g of the Te-FO samples would result in an intake of approximately 10 µg of TAs, respectively. This amount exceeds, near 10 times, the ARfD established by the CONTAM panel for a person of 60 kg, so they can be a risk of acute toxicity for the consumers. Teff flour is used as a substitute in whole or in part for regular flour, mainly for making breads, pancakes and porridge. Despite TAs can be degraded in some extent under some cooking techniques [16], studies in that respect are very scarce, so more studies are needed to clarify the effect of the preparation conditions (time, temperature, etc.) on the final concentration of this toxins. Nevertheless, given the well-known and intense acute toxicity of TAs, more careful scrutiny of TAs contamination in commercial GF products should be enforced.

## 4. Conclusions

In this paper, M-SBA-15-SO_3_^−^ mesostructured silica was employed for the efficient extraction and purification of TAs (atropine and scopolamine) prior to the analysis by HPLC-MS/MS. Samples were firstly treated by SLE and then, the M-SBA-15-SO_3_^−^ was applied as SCX-SPE sorbent. The mesostructured silica was compared with a commercial material and was demonstrated to possess excellent extraction performance. The method validation showed great success with good accuracy, precision, linearity and low MQL. It is important to highlight the advantage of the use of the SPE clean-up stage with the M-SBA-15-SO_3_^−^, such as the reduction of the matrix effect in the analysis. Finally, the method was successfully applied to 15 types of GF pseudocereals, cereals and legumes collected from the market. In 60% of the samples analysed at least one alkaloid was detected. Atropine was found in eight of the samples between, 7 ± 1 and 78 ± 12 µg/kg, while scopolamine was only found in the teff flour sample, being its concentration 28 ± 6 µg/kg. The positive finding of tropane alkaloids in GF foods underlines the importance of the need for sensitive and reliable methods for their analysis and wider studies on atropine and scopolamine occurrence in these types of products.

## Figures and Tables

**Table 1:** Textural properties and functionalization degree of the prepared SBA-15 silicas compared with a commercial sorbent. Recovery percentages for atropine and scopolamine in standard solution using synthetized and commercial materials as strong cation-exchange solid-phase extraction (SCX-SPE) sorbent.

Material	S_BET_^a^ (m^2^/g)	Pore Volume ^b^ (cm^3^/g)	Pore Size ^c^ (Å)	L_0_^d^ (mmol S/g)	SO_3_^−^ Groups ^e^ (mmol/g)	Recovery (% ± SD) ^f^	
						Atropine	Scopolamine
**SBA-15**	**780**	**0.8**	**56**	**-**	-	-	-
L-SBA-15-SO_3_^−^	587	0.7	55	0.5	0.3	38 ± 30	51 ± 13
M-SBA-15-SO_3_^−^	460	0.6	49	0.9	0.5	90 ± 8	91 ± 9
H-SBA-15-SO_3_^−^-	350	0.4	36	1.7	0.6	95 ± 0	104 ± 12
MFE-PAK^®^ SCX	398	0.6	61	0.8	0.3	91 ± 2	91 ± 2

^a^ S_BET_: Specific surface area estimated by Brunauer-Emmett-Teller method. ^b^ Pore volume: Total pore volume measured at relative pressure 0.97. ^c^ Pore size: Diameter of pore estimated by Barret-Joyner-Halenda model applied in the desorption branch. ^d^ L_0_: Degree of functionalization calculated through the sulfur percentage determined by elemental analysis. ^e^ SO_3_^−^ groups: estimated by titration. ^f^ SPE experimental conditions—Sorbent amount: 50 mg. Conditioning: 2 mL of MeOH and 2 mL of 0.025 M phosphate buffer pH 4.8. Loading: 4 mL of 0.5 mg/L TAs standard solution, dissolved in MeOH/0.025 M phosphate buffer pH 4.8 (50:50, *v*/*v*). Washing: 2 mL of 0.025 M phosphate buffer pH 4.8. Elution: 3 × 3 mL of MeOH + 10% ammonia solution pH 11.8.

**Table 2:** Recovery percentages (% ± SD) for atropine and scopolamine in standard solutions (Ss) and buckwheat (Bu-F1O) and sorghum (So-FO) flours extracts using sulfonic acid-functionalized SBA-15 and commercial materials as SCX-SPE sorbents *.

Solvent	M-SBA-15-SO3^−^ SCX-SPE Sorbent						**MFE-PAK^®^ SCX-SPE Sorbent**	
	Atropine			Scopolamine			**Atropine**	**Scopolamine**
	Ss	Bu-F1O	So-FO	Ss	Bu-F1O	So-FO	**Ss**	**Ss**
ACN/H_2_O, 0.5% HCOOH (3:5, *v*/*v*)	74 ± 2	46± 22	10 ± 1	81 ± 4	5 ± 1	8 ± 1	-	-
ACN/H_2_O, 1% HCOOH (3:5, *v*/*v*)	78 ± 1	7 ± 3	7 ± 2	80 ± 5	4 ± 2	7 ± 3	-	-
MeOH/H_2_O, 0.5% HAc (2:1, *v*/*v*)	86 ± 3	16 ± 3	15 ± 2	105 ± 10	12 ± 1	16 ± 0	65 ± 3	65 ± 2
H_2_O, 1.1% HCl (pH 1.0)	90 ± 7	43 ± 10	66 ± 3	103 ± 1	25 ± 7	48± 3	36 ± 4	15 ± 2

* SPE experimental conditions - Sorbent amount: 50 mg. Loading: 8 mL of 0.1 mg/L TAs standard solution or sample extracts from flours spiked with 0.1 mL of a 1 mg/L TAs standard solution. Elution: 3 mL of MeOH and 2 × 3 mL MeOH + 10% of ammonia solution (pH 11.8). Conditioning (5 mL) and washing (3 mL) of the same mixture used for the loading step.

**Table 3:** Recovery percentages (% ± SD) for atropine (At) and scopolamine (Sc) obtained in buckwheat (Bu-F1O), sorghum (So-FO) and chickpea (Ch-FC) flours spiked with 0.1 mL of a 1 mg/L standard solution of atropine and scopolamine under different SLE-SPE conditions, using of sulfonic acid-functionalized SBA-15 and commercial materials as SCX-SPE sorbents *.

Sample	M-SBA-15-SO_3_^−^ SCX-SPE Sorbent									MFE-PAK^®^ SCX SPE Sorbent			
	50 mg		100 mg		150 mg			200 mg		150 mg		200 mg	
	At	Sc	At	Sc	At	Sc	At		Sc	At	Sc	At	Sc
Bu-F1O	43 ± 10	25 ± 7	52 ± 21	33± 17	87 ± 9	70 ± 5	-		-	48 ± 6	10 ± 3	60 ± 16	14 ± 5
So-FO	66 ± 3	48 ± 3	82 ± 16	85 ± 8	98 ± 4	95 ± 4	-		-	59 ± 8	21 ± 1	71 ± 11	34 ± 2
Ch-FC	-	-	60 ± 1	27 ± 3	93 ± 13	41 ± 2	92 ± 9		72 ± 4	39 ± 2	17 ± 4	48 ± 2	20 ± 1

* 1 g of flour was subject to SLE with 8 mL of H_2_O, 1.1% HCl (pH 1.0). SPE experimental conditions as in Table 2.

**Table 4:** Linearity, matrix effect and limits for atropine and scopolamine in different samples.

		Atropine					Scopolamine				
**Sample**	Linearity (mg/L)	Matrix Matched Calibration (R^2^)	Cm * (%)	MDL * (µg/kg)	MQL * (µg/kg)	ME * (%)	Matrix Matched Calibration (R^2^)	Cm (%)	MDL (µg/kg)	MQL (µg/kg)	ME (%)
Co-RFC	0.002–1	8.6 × 10^8^x −8.5 × 10^6^ (0.998)	98	0.1	0.4	17 ^a^	3.3 × 10^8^x + 4.3 × 10^4^ (0.999)	94	0.2	0.7	1 ^d^
So-FO	0.01–0.2	7.1 × 10^8^x −3.9 × 10^6^ (0.996)	98	0.5	1.5	−3 ^a^	3.0 × 10^8^x +6.0 × 10^5^ (0.999)	99	0.7	2.4	−7 ^d^
Te-FO	0.01–0.2	7.0 × 10^8^x −1.24 × 10^7^ (0.994)	92	0.04	0.1	−5 ^a^	2.9 × 10^8^x +3.2 × 10^4^ (0.994)	98	1	3.3	−11 ^d^
Co-FO	0.002–1	8.3 × 10^8^x −5.8 × 10^6^ (0.997)	97	0.1	0.4	14 ^a^	3.1 × 10^8^ −4.92 × 10^6^ (0.996)	96	0.2	0.7	−5 ^d^
Pe-FC	0.01–0.4	5.6 × 10^8^x −1.1 × 10^7^ (0.993)	95	0.3	1.1	−35 ^b^	2.1 × 10^8^x −2.7 × 10^6^ (0.993)	98	1.3	4.4	−51 ^e^
Am-GO	0.01–1	3.62 × 10^8^x +1.18 × 10^6^ (1.000)	96	0.2	0.5	−51 ^c^	1.0 × 10^8^x +1.4 × 10^6^ (0.996)	96	0.5	1.7	−69 ^f^

* Cm: Linearity coefficient. MDL: Method detection limit. MQL: Method quantification limit. ME: Matrix effect. Different superscript letters in the same column indicated significant differences (*p* ≤ 0.05).

## Supplementary Materials

The following are available online at https://www.mdpi.com/2304-8158/9/12/1854/s1. Table S1: Samples analysed in this work; Table S2: Information declared in the nutrition facts label of the samples analysed in this work; Table S3: Instrumental validation parameters of the HPLC/MS-MS separation; Table S4: Evaluation of the matrix effect comparing the spiked sample (0.1 mg/kg) with a standard solution of atropine and scopolamine with the same concentration *; Table S5: Recovery, intraday and interday repeatability of the method calculated on a sorghum flour sample (So-FO) spiked with atropine and scopolamine; Table S6: Textural properties and functionalization degree of five different batches of prepared M-SBA-15-SO_3_^−^ sorbent; Table S7: RASFF notifications of the last five years (2015-2020) on atropine and scopolamine in cereal and bakery products; Figure S1: XRD patterns of: (a) SBA-15; (b) L-SBA-15-SO_3_^−^, (c) M-SBA-15-SO_3_^−^ and (d) H-SBA-15-SO_3_^−^; Figure S2: Pore size distribution of: (a) SBA-15, (b) L-SBA-15-SO_3_^−^, (c) M-SBA-15-SO_3_^−^ and (d) H-SBA-15-SO_3_^−^.
