# Supplementary material for: Sulfonic Acid-Functionalized SBA-15 as Strong Cation-Exchange Sorbent for Solid-Phase Extraction of Atropine and Scopolamine in Gluten-Free Grains and Flours
Source: Foods. 2020 Dec 12;9(12):1854. doi: 10.3390/foods9121854 (PMC7763219; doi:10.3390/foods9121854)
Supplement: Supplementary file 1 [file foods-09-01854-s001.pdf]

# Supplementary material

**Table S1.** Samples analysed in this work.

| Code | Name | Scientific name | Type | Format | Type of farming | Additional Information |
|---|---|---|---|---|---|---|
| **Bu-F1O** | Buckwheat | *Fagopyrum Esculentum* | Pseudo-cereal | Flour | Organic | - |
| **Bu-F2O** | Buckwheat | *Fagopyrum Esculentum* | Pseudo-cereal | Flour | Organic | - |
| **Bu-GC** | Buckwheat | *Fagopyrum Esculentum* | Pseudo-cereal | Grain | Conventional | - |
| **Qu-GO** | Quinoa | *Chenopodium quinoa* | Pseudo-cereal | Grain | Organic | - |
| **Am-GO** | Amaranth | *Amaranthus spp* | Pseudo-cereal | Grain | Organic | - |
| **Te-FO** | Teff | *Eragrostis tef* | Cereal | Flour | Organic | Whole flour |
| **Co-RFC** | Corn | *Zea mays* | Cereal | Flour | Conventional | Refined |
| **Co-FO** | Corn | *Zea mays* | Cereal | Flour | Organic | - |
| **bCo-FC** | Blue corn | *Zea mays* | Cereal | Flour | Conventional | - |
| **So-FO** | Sorghum | *Sorghum bicolor* | Cereal | Flour | Organic | - |
| **Mi-GO** | Millet | *Panicum miliaceum* | Cereal | Grain | Organic | Peeled |
| **rLe-FO** | Red lentil | *Lens culinaris* | Legume | Flour | Organic | - |
| **gLe-FO** | Green lentil | *Lens culinaris* | Legume | Flour | Organic | - |
| **Ch-FC** | Chickpea | *Cicer arietinum* | Legume | Flour | Conventional | - |
| **Pe-FC** | Pea | *Pisum sativum* | Legume | Flour | Conventional | - |

**Table S2.** Information declared in the nutrition facts label of the samples analysed in this work.

| Sample | Per 100 g | | | | | | |
|---|---|---|---|---|---|---|---|
| | **Fat (g)** | **Of which saturated (g)** | **Carbohydrates (g)** | **Of which sugars (g)** | **Fibre (g)** | **Protein (g)** | **Sodium (g)** |
| **Buckwheat *** | 3.2 | 0.66 | 65.9 | 1.16 | 3 | 12.9 | 0.001 |
| **Quinoa** | 5.94 | 0.53 | 62.44 | 1.6 | - | 12.19 | - |
| **Amaranth** | 7 | 1.5 | 65.2 | 1.7 | 6.7 | 13.6 | 0 |
| **Teff** | 3.3 | 0.8 | 60.1 | 3.2 | 5.9 | 8.8 | 0.08 |
| **Refined Corn** | <0.5 | <0.1 | 86 | 0 | 1 | <0.5 | <0.01 |
| **Corn** | 3 | 1 | 66 | 2 | - | 9 | 0.002 |
| **Blue Corn** | 2 | 0.4 | 80.3 | 0 | - | 6.6 | 0 |
| **Sorghum** | 2.6 | 0.4 | 72 | 1.3 | 4.3 | 8.1 | 0.01 |
| **Millet** | 4.2 | 0.72 | 64.3 | 8.2 | 8.5 | 11.2 | 0.01 |
| **Red Lentil** | 2.6 | 0.2 | 54.9 | 2.3 | 5.2 | 27.2 | 0.003 |
| **Green Lentil** | 2.2 | 0.4 | 59.2 | 0.4 | 10.8 | 25 | <0.02 |
| **Chickpea** | 6.5 | 0.8 | 55.7 | 7.4 | 9.67 | 21.33 | <0.1 |
| **Pea** | 1 | 0 | 64 | 0 | 6 | 22 | 0 |

* Buckwheat flour of organic farming sample (Bu-F1O)

**Table S3.** Instrumental validation parameters of the HPLC/MS-MS separation.

| Parameters | Concentrations | Atropine | Scopolamine |
|---|---|---|---|
| **Linear range (µg/mL)** | | 0.005- 5 | 0.005 - 5 |
| **Linearity** | | $7.3 \cdot 10^8x + 1.5 \cdot 10^7$ | $3.3 \cdot 10^8x + 1.8 \cdot 10^7$ |
| **$R^2$** | | 0.998 | 0.996 |
| **Slope RSD%** | | 7 | 2 |
| **LOD[a] (µg/L)** | | 0.004 | 0.014 |
| **LOQ[b] (µg/L)** | | 0.013 | 0.05 |
| **Repeatability, RSD%** | 0.05 µg/L | 2 | 4 |
| | 5 µg/L | 1 | 2 |
| | 200 µg/L | 1 | 1 |
| | 5000 µg/L | 2 | 1 |
| **Whithin-laboratory reproducibility, RSD%** | 0.05 µg/L | 5 | 4 |
| | 5 µg/L | 5 | 8 |
| | 200 µg/L | 5 | 3 |
| | 5000 µg/L | 7 | 7 |

[a] Limit of detection.

[b] Limit of quantification.

**Table-S4.** Evaluation of the matrix effect comparing the spiked sample (0.1 mg/kg) with a standard solution of atropine and scopolamine with the same concentration*.

| | **Atropine** | | |
|---|---|---|---|
| **Sample** | **Group 1** | **Group 2** | **Group 3** |
| Buckwheat | Bu-F1O | | |
| Quinoa | Qu-GO | | |
| Corn | Co-RFC | | |
| Blue corn | bCo-FC | | |
| Corn | Co-FO | | |
| Teff | Te-FO | | |
| Millet | Mi-GO | | |
| Sorghum | So-FO | | |
| Amaranth | | Am-GO | |
| Chickpea | | Ch-FC | |
| Red lentil | | rLe-FO | |
| Pea | | | Pe-FC |
| Green lentil | | | gLe-FO |
| | **Scopolamine** | | |
| **Sample** | **Group 1** | **Group 2** | **Group 3** |
| Corn | Co-RFC | | |
| Buckwheat | Bu-F1O | | |
| Quinoa | Qu-GO | | |
| Sorghum | So-FO | | |
| Corn | Co-RFC | | |
| Millet | Mi-GO | | |
| Teff | Te-FO | | |
| Blue corn | bCo-FC | | |
| Red lentil | | rLe-FO | |
| Chickpea | | Ch-FC | |
| Amaranth | | Am-GO | |
| Green lentil | | | Pe-FC |
| Pea | | | gLe-FO |

* Data were statistically compared by Analysis of Variance (ANOVA) and Duncan's Multiple range test.

**Table S5.** Recovery, intra-day and inter-day repeatability of the method calculated on a sorghum flour sample (So-FO) spiked with atropine and scopolamine.

| Analyte level | Recovery (% ± SD) | Intra-day precision (%RSD) | Inter-day precisión (%RSD) |
|---|---|---|---|
| Atropine (µg/kg) | | | |
| 1.5 | 105 ± 9 | - | - |
| 5 | 93 ± 7 | 4 | 8 |
| 100 | 94 ± 12 | 11 | 13 |
| Scopolamine (µg/kg) | | | |
| 2.4 | 96 ± 8 | - | - |
| 5 | 96 ± 17 | 6 | 18 |
| 100 | 93 ± 17 | 8 | 19 |

**Table S6.** Textural properties and functionalization degree of five different batches of prepared M-SBA-15-$SO_3^-$ sorbent.

| M-SBA-15-$SO_3^-$ | $S_{BET}$ ($m^2/g$)[a] | Pore volume ($cm^3/g$)[b] | Pore size (Å)[c] | $L_0$ (mmol S/g)[d] |
|---|---|---|---|---|
| **Batch 1** | 475.1 | 0.56 | 48.5 | 0.944 |
| **Batch 2** | 461.8 | 0.54 | 49.4 | 0.981 |
| **Batch 3** | 533.6 | 0.61 | 49.5 | 1.049 |
| **Batch 4** | 507.4 | 0.58 | 49.1 | 1.151 |
| **Batch 5** | 459.4 | 0.52 | 50.0 | 0.928 |

[a] $S_{BET}$: Specific surface area calculated by Brunauer-Emmett-Teller (BET) method. [b] Pore volume: Total pore volume measured at relative $P/P_0$=0.97.[c] Pore size: Pore diameter estimated by Barret-Joyner-Halenda (BJH) model applied in the desorption branch. [d] $L_0$: Functionalization degree estimated through the %S determined by elemental analysis.

**Table S7.** RASFF notifications of the last 5 years (2015-2020) on atropine and scopolamine in cereal and bakery products.

| Date | Country | Subject | Action taken | Decision |
|---|---|---|---|---|
| 04/06/2015 | Germany | Atropine (198.5 μg/kg - ppb) and scopolamine (45 μg/kg - ppb) in organic polenta cornmeal from Germany | Recall from consumers | Serious |
| 18/09/2015 | Germany | Atropine (29 μg/kg - ppb) and scopolamine (6 μg/kg - ppb) in microwave popcorn from Spain | Recall from consumers | Serious |
| 26/11/2015 | Czech Republic | Atropine (1200; 1500 μg/kg - ppb) and scopolamine (360; 460 μg/kg - ppb) in sorghum flour from the Czech Republic with raw material from the Czech Republic and Slovakia | Withdrawal from the market | Serious |
| 01/02/2016 | Czech Republic | Atropine (180; 130 μg/kg - ppb) and scopolamine (36;27 μg/kg - ppb) in gluten-free baking mix and related products based on sorghum from the Czech Republic | Destruction | Serious |
| 13/04/2016 | Czech Republic | Atropine (7.9 μg/kg - ppb) in baby porridge without milk from Spain | Withdrawal from the market | Undecided |
| 22/07/2016 | Austria | Atropine (10.5; 12.2; 12.3 μg/kg - ppb) and scopolamine (2.0; 3.8; 3.1 μg/kg - ppb) in corn from Germany | Withdrawal from the market | Serious |
| 20/09/2016 | Austria | Atropine (23.5 μg/kg - ppb) and scopolamine (9.5 μg/kg - ppb) in millet flour from Hungary | Public warning - press release | Serious |
| 24/05/2018 | Germany | Atropine (6.60 μg/kg - ppb) and scopolamine (1.77 μg/kg - ppb) in popcorn from France | Withdrawal from the market | Serious |
| 05/06/2018 | Germany | Scopolamine (10.3 μg/kg - ppb) in popcorn in grain from Argentina | Recall from consumers | Serious |
| 26/09/2018 | Germany | Atropine (60.7 μg/kg - ppb) and scopolamine (38.8 μg/kg - ppb) in organic muesli from Austria | Recall from consumers | Serious |
| 20/12/2018 | France | Atropine (53 μg/kg - ppb) and scopolamine (20 μg/kg - ppb) in organic buckwheat flour from France | | Serious |
| 01/04/2019 | Croatia | Atropine (4.5 μg/kg - ppb) and scopolamine (4.3 μg/kg - ppb) in corn grits from Serbia | Public warning - press release | Serious |
| 06/08/2019 | Netherlands | Atropine (2.2 μg/kg - ppb) and scopolamine (2.7 μg/kg - ppb) in breakfast porridge 8 cereals with honey from Spain | Withdrawal from the market | Undecided |
| 26/08/2019 | France | Atropine (47 μg/kg - ppb) and scopolamine (30 μg/kg - ppb) in organic buckwheat flour from France | Recall from consumers | Serious |
| 24/01/2020 | Germany | Atropine (19 μg/kg - ppb) and scopolamine (6.4 μg/kg - ppb) in organic soy flakes from Austria, with raw material from the Czech Republic | Withdrawal from the market | Serious |

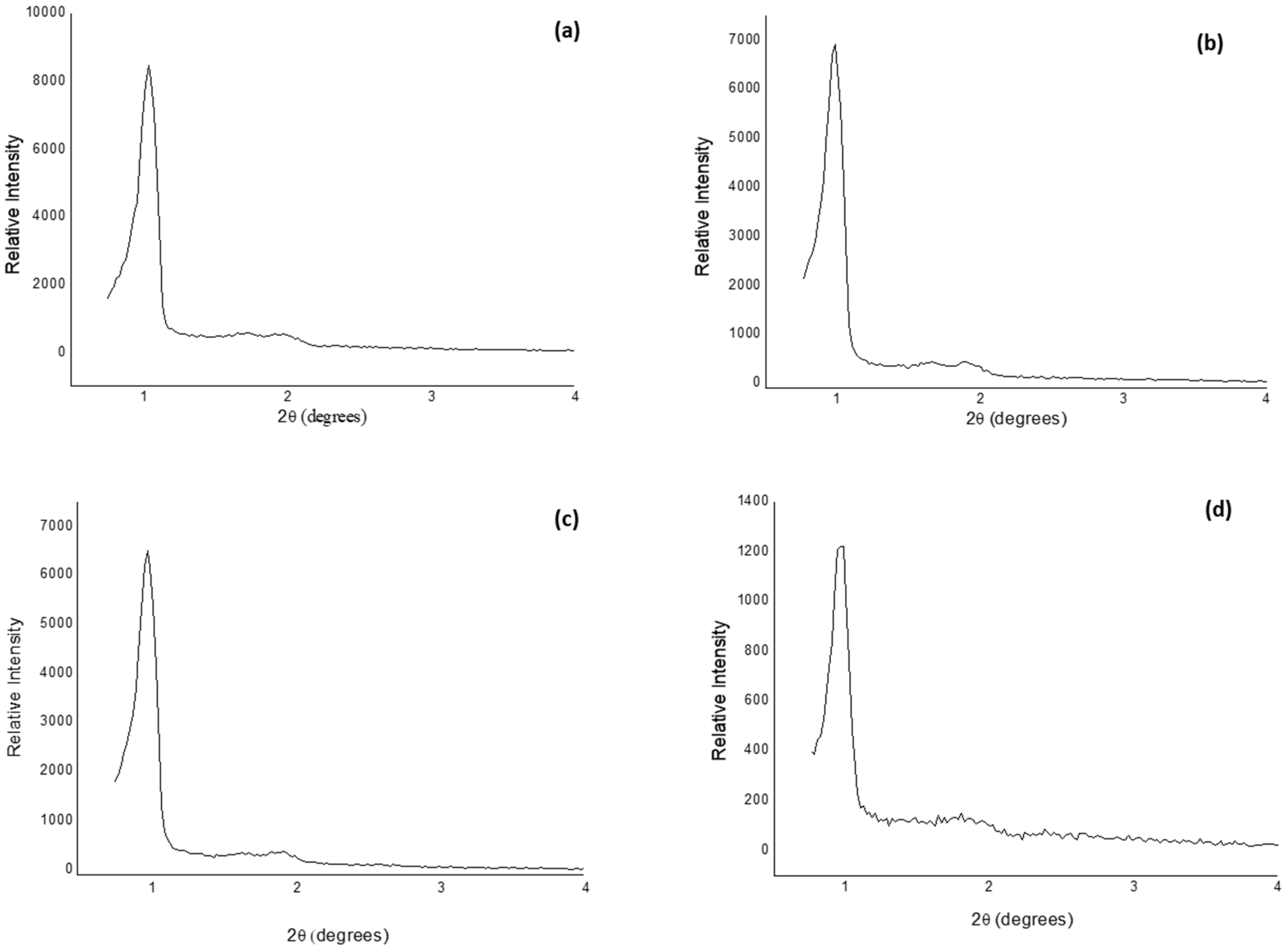


**Figure S1.** XRD patterns of: (a) SBA-15; (b) L-SBA-15-$SO_3^-$, (c) M-SBA-15-$SO_3^-$ and (d) H-SBA-15-$SO_3^-$.

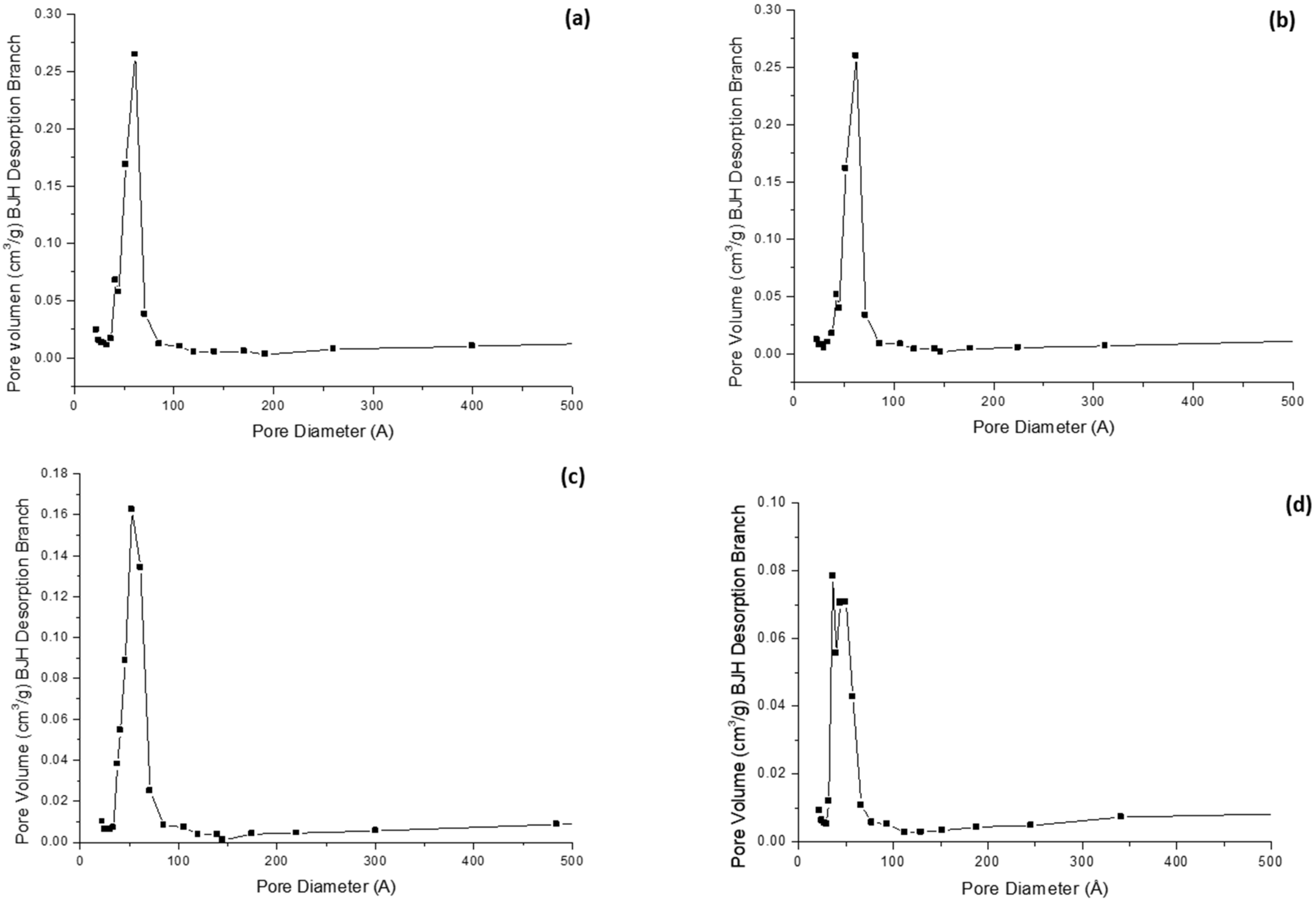


**Figure S2.** Pore size distribution of: (a) SBA-15, (b) L-SBA-15-$SO_3^-$, (c) M-SBA-15-$SO_3^-$ and (d) H-SBA-15-$SO_3^-$.
